# Integrating whole-genome sequencing and metagenomics improves phenotype prediction of fat deposition traits in pigs

**DOI:** 10.1186/s12711-026-01116-y

**Published:** 2026-11-13

**Authors:** Yiyi Liu, Meng Lin, Jun Luo, Jie Yang, Zekai Yao, Danyang Lin, Haiqi Qiu, Zebin Zhang, Sixiu Huang, Enqin Zheng, Gengyuan Cai, Zhenfang Wu, Langqing Liu, Jia-Jin Wu

**Affiliations:** 1 State Key Laboratory of Swine and Poultry Breeding Industry, Guangdong Laboratory for Lingnan Modern Agriculture, National Engineering Research Center for Breeding Swine Industry, and College of Animal Science, South China Agricultural University, Guangzhou, Guangdong China; 2 National and Regional Livestock Genebank, Guangdong Gene Bank of Livestock and Poultry, South China Agricultural University, Guangzhou, Guangdong China; 3 Guangdong Zhongxin Breeding Technology Co., Ltd, Guangzhou, Guangdong China; 4 Wens Foodstuff Group Co., Ltd., Yunfu Subcenter of Guangdong Laboratory for Lingnan Modern Agriculture, Yunfu, Guangdong China

## Abstract

**Background:**

Fat deposition is a key economic trait affecting production efficiency and carcass quality in pigs and is influenced by both genetic and environmental factors. Although increasing evidence indicates that the gut microbiome contributes to host phenotypic variation, current genomic evaluation and prediction models largely rely on host genotype information and rarely account for microbiome effects. This study aimed to quantify the contribution of the gut microbiome to fat deposition traits and to evaluate whether incorporating microbiome information could improve phenotypic prediction in pigs.

**Results:**

We collected phenotypic characteristics of six fat deposition traits including the shoulder backfat thickness, the 6th-7th rib backfat thickness, the last rib backfat thickness, waist backfat thickness, the 3rd-4th last rib backfat thickness, and the average backfat thickness from 478 Duroc × Landrace × Yorkshire pigs. Using whole-genome resequencing and metagenomic sequencing, we identified 21 million single nucleotide variants and quantified the abundances of 20,336 gut microbial species across 478 pigs. Distinct fecal microbial community structures were observed between pigs with high- and low-fat deposition, and 27 bacterial species were identified as potential biomarkers associated with fat deposition. Microbiability estimates for the six backfat thickness traits ranged from 0.17 to 0.44, indicating that the gut microbiome accounted for a considerable proportion of phenotypic variance beyond host genetic contributions. In contrast, the interaction effects between the host genome and gut microbiome accounted for only a negligible proportion of phenotypic variance across the six traits. Incorporating overall microbiome information into genomic prediction models resulted in mean phenotypic predictive abilities ranging from 0.082 to 0.292, representing increases in predictive ability of 0.023 to 0.084 across traits and reductions in the mean squared error of prediction of 0.001 to 0.009 compared with the additive genetic model.

**Conclusions:**

Our findings demonstrate that the gut microbiome makes a substantial contribution to variation in fat deposition traits in pigs. Integrating host whole-genome resequencing and gut metagenomic information yielded modest absolute gains in phenotypic predictive ability, suggesting that the gut microbiome may provide complementary information for prediction models of complex traits in pigs.

**Supplementary Information:**

The online version contains supplementary material available at https://doi.org/10.1186/s12711-026-01116-y.

## Background

Pork is the most widely consumed meat globally and is valued as a high quality protein source [1]. Subcutaneous fat deposition, commonly proxied by backfat thickness (BF), directly affects production efficiency and carcass quality. Excessive backfat reduces carcass lean percentage [2] and is genetically associated with poorer feed efficiency, as indicated by a higher feed conversion ratio [3]. Thus, targeted genetic improvement of backfat thickness holds substantial practical significance for the swine industry, with direct benefits for production efficiency, carcass quality, and long-term sustainability.

Genomic selection, also referred to as genomic prediction, is a modern breeding method that utilizes genome-wide marker information to estimate the genomic estimated breeding values (GEBV) of individuals. By enabling animals to be evaluated early in life without requiring their own phenotypic records, genomic selection can increase the accuracy of genetic evaluation, shorten the generation interval, and accelerate genetic gain in livestock breeding programs [4]. Currently, most research on pigs has focused on identifying host genetic variants that affect fat deposition [5], and genomic prediction models largely rely solely on host genomic data. It is important to note that an animal is a holobiont, a symbiotic entity comprising the host organism and its microbiome. The expression of complex host traits is not only regulated by the host’s own genetics but also by its gut microbiota. Studies in humans [6], mice [7], and livestock [8–10] have reported associations between the gut microbiota and obesity or fat deposition. For instance, differences in the relative abundance of Firmicutes and Bacteroidetes have been observed between obese and lean individuals [11]. However, whether the association between these phyla and obesity represents a consistent microbial signature remains controversial across studies [12]. Pilot study in pig shows that specific gut bacteria modulate fat deposition. *Escherichia spp.* are consistently enriched in the jejunum, ileum and cecum locations of high-fat deposition pigs [13]. Additionally, *Lactobacillus reuteri* promotes intramuscular fat deposition by enhancing branched-chain amino acid (BCAA) synthesis [14]. In contrast, *Eubacterium siraeum* appears to inhibit adipogenesis by suppressing the PI3K/AKT signaling pathway [15]. These findings underscore the gut microbiota’s role as a key modulator of fat deposition.

Inspired by the quantitative genetic concept of heritability, the “microbiability” has been proposed to quantify the proportion of host phenotypic variation explained by microbiota [16]. Applying this framework, studies across species consistently significant and largely independent contributions of microbiota to complex traits [17–19]. Collectively, these findings position gut microbiota data as a powerful and complementary resource for prediction of complex traits. Furthermore, metagenomics and other multi-omics approaches not only provide novel predictive perspectives but also reveal phenotype-genotype associations elusive to genomic sequencing alone [20]. Existing studies have demonstrated that incorporating such multi-omics data into prediction models can improve genetic evaluation and prediction accuracy [21–23].

In this study, the aim was to investigate the relationship between host genetics, gut microbiota, and fat deposition traits in Duroc × Landrace × Yorkshire (DLY) pigs. Specifically, the objectives of this study were to: (i) characterize gut microbiome features associated with fat deposition traits in DLY pigs; (ii) estimate the contributions of host genetic effects, gut microbiome effects, and their interaction to variation in fat deposition traits; and (iii) evaluate whether incorporating gut microbiome information can improve the phenotypic predictability of fat deposition traits. This study provides a basis for understanding the contribution of gut microbiome to fat deposition and for exploring the phenotypic prediction models that incorporate microbiome information.

## Methods

### Animals, sample collection, and phenotypic data

A total of 478 Duroc × Landrace × Yorkshire (DLY) pigs were included in this study. All animals, provided by the Wen’s Foodstuff Group Co., Ltd. (Guangdong, China), were raised under standardized management with ad libitum access to a common feed and water. Ear tissue samples were collected and stool samples were obtained by rectal swabbing at approximately 110 ± 5 kg. All samples were snap-frozen in liquid nitrogen and stored at -80 °C for subsequent studies. Thereafter, all pigs were slaughtered following standard procedures.

Backfat thickness at different anatomical sites was measured using a vernier caliper, including the shoulder backfat thickness (BF1), the 6th–7th rib backfat thickness (BF2), the last rib backfat thickness (BF3), waist backfat thickness (BF4), and the 3rd–4th last rib backfat thickness (BF5). The average backfat thickness (BF6) was expressed as the mean of shoulder backfat, the last rib backfat, and waist backfat to evaluate the uniformity of dorsal backfat. After collecting phenotypic data, the raw phenotypic records for each trait were adjusted using a single-trait animal model in the BLUPF90 programs. The model used for correction included sex (two levels, male: female = 1.03), farm (four levels), batch (thirteen levels) as fixed effects, and age (176–229 days) as a covariate. The rMVP package [24] was used to visualize the phenotypic distribution of the population.

### Whole‑genome resequencing and data processing

The host genomic DNA was isolated from ear tissue samples following the traditional phenol/chloroform method. A total of 478 host DNA samples were sequenced using an Illumina HiSeq 2500 Sequencer (Illumina, Inc., San Diego, CA, USA) to generate 150 bp paired-end reads. The clean reads were mapped to the pig reference genome (*Sus scrofa* 11.1) using BWA (version 0.7.12) [25] and sorted with Samtools (version 1.9) [26]. Duplicates were removed using Picard tools, and SNP calling and genotyping were performed using the HaplotypeCaller module in the Genome Analysis Toolkit (GATK, version 4.0.2.1) [27]. GATK VariantFiltration was used to remove low-quality variants based on variant-level quality metrics, including quality by depth (QD < 2.0), strand bias (FS > 60.0), mapping quality (MQ < 40.0), mapping quality rank sum test (MQRankSum < − 12.5), and read position rank sum test (ReadPosRankSum < -8.0). Finally, the annotated SNP data were filtered using PLINK (version 1.90) [28] for sample- and marker-level quality control, with the following parameters: sample call rate > 90%, SNP call rate > 90%, and minor allele frequency > 1%. A total of 21,712,014 SNPs were retained for subsequent analysis.

### Metagenomic sequencing and analysis

Total microbial DNA was extracted from stool samples using the TIANamp stool DNA kit and subjected to short-read sequencing (2 $\:\times\:$ 150 bp) with the Illumina HiSeq 2500 platform. The average sequencing depth was 6 Gb per sample. The raw reads were processed using FastQC (http://www.bioinformatics.babraham.ac.uk/projects/fastqc/) for quality control including the removed trimmed splice sequences. Then, the clean reads were aligned individually to the Swine reference genome (*Sus scrofa* 11.1) using KneadData (version 0.5.1) [29], and the host genome reads on the alignment were filtered to obtain microbial genome reads. The unmapped metagenomic reads were assembled into contigs by MEGAHIT (version 1.2.9) [30] for all individuals, and reads from each sample were aligned to the assembled contigs using Bowtie2 (version 2.4.4) [31] to screen for non-assembled reads. All unassembled reads were then co-assembled using MEGAHIT to improve data utilization. Based on the assembled contigs, MetaGeneMark [32] was used with default parameters to predict open reading frames (ORFs), and CD-HIT (version 4.8.1) [33] was used to remove redundancy (95% identity and 90% coverage), resulting in a nonredundant gene catalog. Using DIAMOND software (version 0.9.95) [34], the nonredundant sequences were aligned to the NCBI non-redundant protein database (e-value < 1e-5). Salmon software (version 1.5.1) [35] was used for gene quantification and TPM values of genes were calculated. From the gene abundance table, information on the abundance of a single sample at each taxonomic level (phylum, order, family, genus, species) was obtained. For a given species, the abundance of a given sample was equal to the sum of the annotated gene abundances for that species. Only species with a detection rate of more than 50% were retained. A total of 20,336 bacterial species, encompassing 3,475 genera across 150 phyla, were used for subsequent analysis. The species-level relative abundance matrix contained 478 samples and 20,336 microbial species and the proportion of zero values in this matrix was 12.41%.

### Statistical analysis

Following adjustment of average backfat thickness (BF6) for the fixed effects of sex, farm, batch, and age, individuals were ranked according to their adjusted BF6 values. The top and bottom 10% of pigs were assigned to the high-backfat and low-backfat groups, respectively (47 individuals per group). The dominant bacterial taxa in each group were subsequently characterized based on their relative abundances at different taxonomic levels. The alpha and beta diversity were assessed based on species-level profiles. Alpha diversity indices, including Shannon index and Simpson index, were calculated using the diversity function from the vegan package. The significance of differences in alpha diversity between groups was verified by the Wilcoxon rank-sum test, with a significance threshold of 0.05. The beta diversity was evaluated by computing Bray-Curtis distances with the “vegdist” function from the vegan package, and Principal co-ordinates analysis (PCoA) was used to achieve dimensionality reduction of data. Permutational multivariate analysis of variance (PERMANOVA) was used to quantify the explanatory power of group effects on community structure variation based on 999 permutation tests [36]. To identify characteristic microbial markers significantly associated with fat deposition, the Meta-analysis via Mixed Models Utilizing Public Health Information (MMUPHin) [37] was used to correct for the effects of sex, farm, and age on the gut microbiota. All microbiome samples collected from the pigs were processed and sequenced in the same batch. The Linear discriminant analysis effect size (LEfSe) was applied in the microeco package [38]. A linear discriminant analysis Score (LDA Score) greater than 2 was set as the screening threshold for characteristic microorganisms. Statistical analysis and visualization were implemented in R v4.2.3.

### Matrix construction

The genetic relationship matrix (GRM) was constructed using GCTA software (version 1.93.2) [39] based on the following equation:

1 $$\:\begin{array}{c}{g}_{ij}=\frac{1}{N}\sum\limits_{v=1}^{N}\frac{\left({x}_{iv}-2\bar{{p}_{v}}\right)\left({x}_{jv}-2\bar{{p}_{v}}\right)}{2\bar{{p}_{v}}(1-\bar{{p}_{v}})}\mathrm{,}\end{array}$$

where $\:{g}_{ij}$ represents the genetic relationship between individuals *i* and *j*; $\:{x}_{iv}$ and $\:{x}_{jv}$ are the number of reference alleles in pigs *i* and *j*, respectively; $\:{p}_{v}$ represents the reference allele frequency; and *N* is the total number of SNPs.

To assess the proportion of variation in fat deposition explained by the gut microbiome, a microbial relationship matrix (MRM) was constructed using the species abundance table according to the equation described by Wen et al. [17]:

2 $$\:\begin{array}{c}{m}_{ij}=\frac{1}{N}\sum\limits_{k=1}^{N}\frac{({x}_{ik}-\bar{{x}_{k}})({x}_{jk}-\bar{{x}_{k}})}{{\sigma\:}_{k}^{2}}\mathrm{,}\end{array}$$

where $\:{m}_{ij}$ represents the estimated value of microbial similarity between pigs *i* and *j*; $\:{x}_{ik}$ and $\:{x}_{jk}$ are the relative abundance of species *k* in pigs *i* and *j*, respectively; $\:\overline{{x}_{k}}$ is the average relative abundance of species *k* in the feces of the whole population; $\:{\sigma\:}_{k}^{2}$ is the variance of relative abundance of species *k*; and *N* is the total number of species.

The correlation between GRM and MRM was evaluated using a Mantel permutation test. Pearson’s correlation coefficient was calculated between the corresponding off-diagonal elements of the two relationship matrices. Statistical significance was assessed using 999 unrestricted permutations of the rows and corresponding columns of one matrix. The analysis was conducted using the “mantel” function implemented in the vegan package.

### Evaluating effects of host genetics and the gut microbiome on fat deposition

To estimate the contributions of host genomic and microbiome effects to variation in fat deposition traits, three linear mixed models were fitted using restricted maximum likelihood in HIBLUP software (version 1.5.3) [40]. Model [A] included the host genomic effect, Model [B] included both host genomic and microbiome effects, and Model [C] further included the interaction effect between the host genome and the gut microbiome. Model [A], including the host genomic effect, was specified as follows:

A $$\:\begin{array}{c}y=Kc+{\mathbf{Z}}_{\mathrm{g}}g+e\mathrm{,}\end{array}$$

Where $\:\mathbf{y}$ is the vector of observations for fat deposition traits; $\:\mathbf{c}$ is the vector of fixed effects (sex, farm, batch, and age), $\:\mathbf{K}$ is the incidence matrix of $\:\mathbf{c}$; $\:\mathbf{e}$ is the vector of residual effects following a distribution of $\:N(0,\mathbf{I}{\sigma\:}_{e}^{2})$, where $\:\mathbf{I}$ is an identity matrix and $\:{\sigma\:}_{e}^{2}$ is the residual variance; and $\:\mathbf{g}$ is the vector of additive genetic effects following a distribution of $\:N(0,\mathbf{G}{\sigma\:}_{g}^{2})$, where $\:\mathbf{G}$ is the GRM and $\:{\sigma\:}_{g}^{2}$ is the additive genetic variance; $\:{\mathbf{Z}}_{\mathrm{g}}$is the incidence matrix relating to additive genetic effects. The heritability was estimated as $\:{\sigma\:}_{g}^{2}/{\sigma\:}_{p}^{2}$, where $\:{\sigma\:}_{p}^{2}$ is the phenotypic variance.

To further quantify the contribution of the gut microbiome to phenotypic variation, Model [A] was extended by including a microbiome effect based on the MRM:

B $$\:\begin{array}{c}y=Kc+{\mathbf{Z}}_{\mathrm{g}}g+{\mathbf{Z}}_{\mathrm{m}}m+e\mathrm{,}\end{array}$$

where $\:\mathbf{m}$ is the vector of gut microbiota effects following a distribution of $\:N(0,\mathbf{M}{\sigma\:}_{m}^{2})$, where $\:\mathbf{M}$ is the MRM and $\:{\sigma\:}_{m}^{2}$ is the microbiome variance; $\:{\mathbf{Z}}_{\mathrm{m}}$ is the incidence matrix relating to microbiome effects. The remaining parameters of the model are as described in Model [A]. The microbiability was estimated as $\:{\sigma\:}_{m}^{2}/{\sigma\:}_{p}^{2}$.

To evaluate whether the interaction between the host genome and the gut microbiome explained additional phenotypic variation, Model [B] was further extended by including an interaction effect:

C $$\:\begin{array}{c}y=Kc+{\mathbf{Z}}_{\mathrm{g}}g+{\mathbf{Z}}_{\mathrm{m}}m+{\mathbf{Z}}_{\mathrm{i}}i+e\mathrm{,}\end{array}$$

where $\:\mathbf{i}$ is the vector of interaction effect between the genetics and gut microbiome, and $\:{\mathbf{Z}}_{\mathrm{i}}$ is the incidence matrix relating to the interaction effects. The interaction effects were assumed to follow a normal distribution $\:\mathbf{i}\sim N(0,\left[\mathbf{G}\odot\mathbf{M}\right]{\sigma}_{i}^{2})$, where $\:{\sigma\:}_{i}^{2}$ is the variance of the interaction between the genetics and gut microbiome, and $\:\mathbf{G}\odot\mathbf{M}$ represents the Hadamard product of the GRM and the MRM.

### Estimation of genetic correlations

Pairwise genetic correlations between traits were estimated using bivariate linear mixed models fitted by restricted maximum likelihood in HIBLUP software (version 1.5.3). For each pair of traits (trait X and trait Y), the model was fitted as:

3 $$\:\begin{aligned}\left(\begin{array}{c}{\mathbf{y}}_{\mathrm{X}}\\\:{\mathbf{y}}_{\mathrm{Y}}\end{array}\right)=&\left(\begin{array}{cc}{\mathbf{K}}_{\mathrm{X}}&\:0\\\:0&\:{\mathbf{K}}_{\mathrm{Y}}\end{array}\right)\left(\begin{array}{c}{\mathbf{c}}_{\mathrm{X}}\\\:{\mathbf{c}}_{\mathrm{Y}}\end{array}\right)\\&+\left(\begin{array}{cc}{\mathbf{Z}}_{\mathrm{g}\mathrm{X}}&\:0\\\:0&\:{\mathbf{Z}}_{\mathrm{g}\mathrm{Y}}\end{array}\right)\left(\begin{array}{c}{\mathbf{g}}_{\mathrm{X}}\\\:{\mathbf{g}}_{\mathrm{Y}}\end{array}\right)\\&+\left(\begin{array}{c}{\mathbf{e}}_{\mathrm{X}}\\\:{\mathbf{e}}_{\mathrm{Y}}\end{array}\right)\mathrm{,}\end{aligned}$$

where $\:{\mathbf{y}}_{\mathrm{X}}$ and $\:{\mathbf{y}}_{\mathrm{Y}}$ are the vectors of observations for trait X and trait Y, respectively; $\:{\mathbf{c}}_{\mathrm{X}}$ and $\:{\mathbf{c}}_{\mathrm{Y}}$ are the corresponding vectors of fixed effects (sex, farm, batch, and age); $\:{\mathbf{K}}_{\mathrm{X}}$ and $\:{\mathbf{K}}_{\mathrm{Y}}$ are the incidence matrices for fixed effects; $\:{\mathbf{g}}_{\mathrm{X}}$ and $\:{\mathbf{g}}_{\mathrm{Y}}$ are the additive genetic effects; $\:{\mathbf{Z}}_{\mathrm{g}\mathrm{X}}$ and $\:{\mathbf{Z}}_{\mathrm{g}\mathrm{Y}}$ are the corresponding incidence matrices; and $\:{\mathbf{e}}_{\mathrm{X}}$ and $\:{\mathbf{e}}_{\mathrm{Y}}$ are the residual effects. The covariance structures of the additive genetic and residual effects were defined as:

4 $$\:\begin{array}{c}Var\left(\begin{array}{c}{\mathbf{g}}_{\mathrm{X}}\\\:{\mathbf{g}}_{\mathrm{Y}}\end{array}\right)=\left(\begin{array}{cc}{\sigma\:}_{gX}^{2}\mathbf{G}&\:{\sigma\:}_{gXY}\mathbf{G}\\\:{\sigma\:}_{gXY}\mathbf{G}&\:{\sigma\:}_{gY}^{2}\mathbf{G}\end{array}\right)\mathrm{,}\end{array}$$

and

5 $$\:\begin{array}{c}Var\left(\begin{array}{c}{\mathbf{e}}_{\mathrm{X}}\\\:{\mathbf{e}}_{\mathrm{Y}}\end{array}\right)=\left(\begin{array}{cc}{\sigma\:}_{eX}^{2}\mathbf{I}&\:{\sigma\:}_{eXY}\mathbf{I}\\\:{\sigma\:}_{eXY}\mathbf{I}&\:{\sigma\:}_{eY}^{2}\mathbf{I}\end{array}\right)\mathrm{,}\end{array}$$

where $\:\mathbf{G}$ is the GRM, and $\:\mathbf{I}$ is an identity matrix. The genetic correlation was calculated as follows:

6 $$\:\begin{array}{c}{r}_{g}=\frac{{\sigma\:}_{gXY}}{\sqrt{{\sigma\:}_{gX}^{2}{\sigma\:}_{gY}^{2}}}\mathrm{,}\end{array}$$

where $\:{r}_{g}$ is the additive genetic correlation coefficient between trait X and trait Y. $\:{\sigma\:}_{gX}^{2}$ and $\:{\sigma\:}_{gY}^{2}$ are the additive genetic variances of trait X and trait Y, respectively. $\:{\sigma\:}_{gXY}$ is the additive genetic covariance between the two traits. The phenotypic correlation was calculated as follows:

7 $$\:\begin{array}{c}{r}_{p}=\frac{{\sigma\:}_{gXY}+{\sigma\:}_{eXY}}{\sqrt{({\sigma\:}_{gX}^{2}+{\sigma\:}_{eX}^{2})\times\:({\sigma\:}_{gY}^{2}+{\sigma\:}_{eY}^{2})}}\mathrm{,}\end{array}$$

where $\:{r}_{p}$ is the phenotypic correlation coefficient between trait X and trait Y. $\:{\sigma\:}_{eX}^{2}$ and $\:{\sigma\:}_{eY}^{2}$ are the residual variances of trait X and trait Y, respectively. $\:{\sigma\:}_{eXY}$ is the residual covariance between the two traits.

### Genomic prediction

In this study, the genomic best linear unbiased prediction (GBLUP) model [41] was fitted using the same random-effect structure as Model [A], with the corrected phenotypic values used as response variables. Similarly, the GMBLUP model, which integrated host genomic and microbiome information, was fitted using the same random-effect structure as Model [B]. For GBLUP, the GRM was used for prediction, whereas in GMBLUP, both the GRM and the MRM were used.

Prediction performance of the model was evaluated by five-fold cross validation. Specifically, all individuals were randomly divided into five subsets. In the training phase, four subsets were selected as the reference group to fit the prediction model, and the remaining subset was used as the validation group. When fitting each training model, the corrected phenotypic values of individuals in the validation population were masked and treated as missing, whereas the relationship matrices required by each model were retained for all individuals. This procedure was repeated until each subset had been used once as the validation population, and the entire five-fold cross-validation process was repeated five times. For each fold, predictive ability was calculated as the Pearson correlation coefficient between the corrected phenotypic values and the predicted random effects in the validation population. The mean-squared error of prediction (MSEP) was calculated as the mean squared difference between the corrected phenotypic values and the corresponding predicted phenotypic values in the validation population. For each replicate, predictive ability and MSEP were first calculated for each of the five folds and then averaged across folds. The final prediction performance of each model was obtained by averaging the five replicate-level means.

## Results

### Distributions and estimation of genetic parameters for fat deposition in pigs

**Fig. 1:**
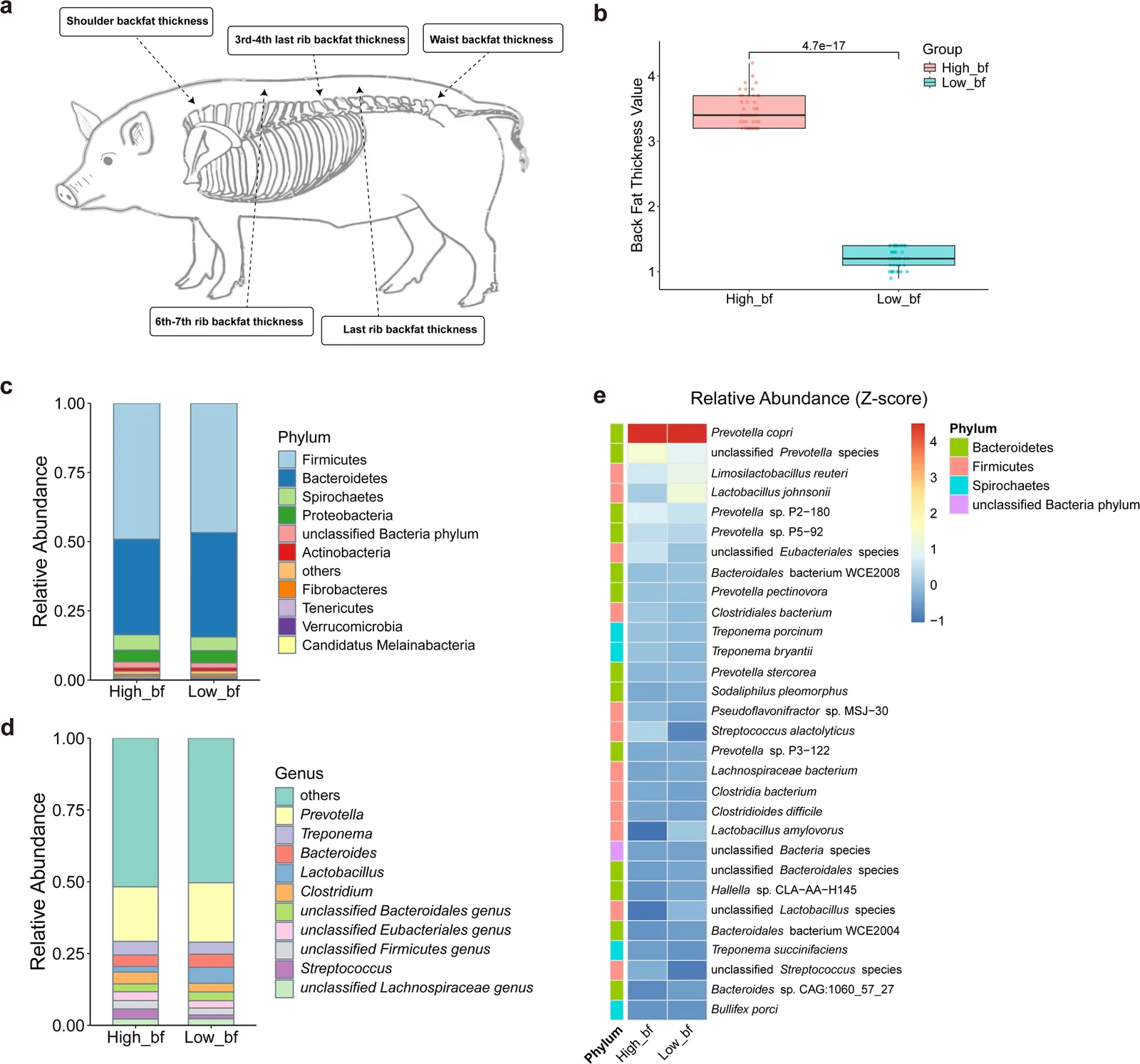
The phenotypic values, and community composition in high- and low- fat deposition pigs. (**a**) Measurement locations for backfat thickness in pigs (**b**) Phenotypic value in high- and low-bf groups. (**c**) Community composition at the phylum level in high- and low-bf groups. (**d**) Community composition at the genus level in high- and low-bf groups. (**e**) Community composition at the species level in high- and low-bf groups. Groups are coded according to the fat deposition status (High_bf, high fat deposition; Low_bf, low fat deposition)

Backfat thickness was measured at five locations on 478 DLY pigs (Fig. 1a), and the average backfat thickness was also considered a phenotypic trait for fat deposition. The mean value of BF1 was the greatest (3.25 cm), whereas that of BF4 was the smallest (1.61 cm). Phenotypic distributions for all traits were consistent with a normal distribution (Additional file 1, Figure S1). All traits showed phenotypic variation, with coefficients of variation ranging from 25.34% for BF1 to 38.44% for BF4 (Table 1).

**Table 1:** Descriptive statistics of phenotypic data on fat deposition traits of DLY pigs

Trait	Unit	Mean	SD	Max	Min	CV (%)
BF1	cm	3.25	0.82	5.70	1.20	25.34
BF2	cm	2.60	0.75	4.80	0.80	28.73
BF3	cm	1.61	0.56	3.30	0.20	35.05
BF4	cm	2.01	0.77	4.10	0.30	38.44
BF5	cm	1.88	0.62	3.70	0.50	32.75
BF6	cm	2.29	0.66	4.20	0.90	28.69

BF1 represents shoulder backfat thickness; BF2 represents the 6th-7th rib backfat thickness; BF3 represents the last rib backfat thickness; BF4 represents waist backfat thickness; BF5 represents the 3rd–4th last rib backfat thickness; BF6 represents the average backfat thickness

The genetic parameters of six fat deposition traits were estimated using whole-genome resequencing data. The heritability estimates for the six BF traits were low to moderate, ranging from 0.13 to 0.38, with the highest estimate observed for BF1 (Table 2). The six BF traits were positively correlated with each other, with genetic correlation coefficients ranging from 0.59 to 1.00 (Table 2). Among them, BF2 showed high genetic correlations with BF1, BF4 and BF6, while the lowest genetic correlation was observed between BF1 and BF3 (0.59 ± 0.18), indicating the heterogeneity of the genetic basis of backfat thickness at shoulder and last rib. Overall, BF6 showed a high genetic correlation with the other BF traits. Except for its correlation with BF3 (0.85 ± 0.07), the correlations between BF6 and the other BF traits were all above 0.90, ranging from 0.90 ± 0.06 to 1.00 ± 0.17 (Table 2). Given that BF6 represents the average backfat thickness and was strongly correlated with backfat thickness at different locations, pigs were subsequently grouped according to their BF6 values.

**Table 2:** Heritability, genetic and phenotypic correlation coefficients among the fat deposition related traits

Trait	BF1	BF2	BF3	BF4	BF5	BF6
BF1	0.38 ± 0.14	0.80 ± 0.03	0.77 ± 0.04	0.65 ± 0.04	0.61 ± 0.03	0.67 ± 0.02
BF2	0.99 ± 0.28	0.13 ± 0.12	0.86 ± 0.04	0.64 ± 0.04	0.63 ± 0.03	0.57 ± 0.03
BF3	0.59 ± 0.18	0.71 ± 0.24	0.36 ± 0.13	0.83 ± 0.03	0.70 ± 0.04	0.59 ± 0.03
BF4	0.68 ± 0.17	1.00 ± 0.41	0.79 ± 0.11	0.33 ± 0.13	0.75 ± 0.03	0.65 ± 0.02
BF5	0.75 ± 0.14	0.90 ± 0.17	0.70 ± 0.14	0.97 ± 0.06	0.29 ± 0.13	0.88 ± 0.03
BF6	0.92 ± 0.05	1.00 ± 0.17	0.85 ± 0.07	0.93 ± 0.04	0.90 ± 0.06	0.37 ± 0.13

BF1 represents shoulder backfat thickness; BF2 represents the 6th-7th rib backfat thickness; BF3 represents the last rib backfat thickness; BF4 represents waist backfat thickness; BF5 represents the 3rd-4th last rib backfat thickness; BF6 represents the average backfat thickness. Values are presented as estimates ± standard errors. Diagonal elements indicate SNP-based heritability estimates, values above the diagonal indicate phenotypic correlations, and values below the diagonal indicate genetic correlations

### Comparison of microbial composition and diversity between the high- and low-fat deposition groups

Among the 478 pigs, we defined individuals in the top 10% and bottom 10% of average backfat thickness (BF6) among the 478 pigs as the high–backfat (High_bf) and low–backfat (Low_bf) groups, respectively. Therefore, 47 pigs with the highest fat deposition (BF6 value: 3.46 ± 0.26 cm) and 47 pigs with the lowest fat deposition (BF6 value: 1.22 ± 0.16 cm) were selected for this study. The BF6 value was significantly different between the high- and low-bf groups (*p*-value < 0.001, Fig. 1b). Based on the microbial taxonomic annotation, at the phylum level, Firmicutes, Bacteroidetes, Spirochaetes, and Proteobacteria were the predominant phyla in the fecal microbiota of DLY pigs (Fig. 1c). Among these, Firmicutes and Bacteroidetes collectively account for 83.9% of the total community. Firmicutes exhibited a higher proportion in the high-bf group than in the low-bf group (49.1% vs. 46.7%), while Bacteroidetes showed the opposite pattern (34.5% vs. 37.7%). At the genus level, *Prevotella*, *Treponema*, *Bacteroides*, *Lactobacillus*, and *Clostridium* were the top five most abundant bacterial genera in both High_bf and Low_bf groups, with mean relative abundances of 19.8%, 4.5%, 4.3%, 3.7%, and 3.6%, respectively. *Prevotella* accounted for 18.9% of the community in the high_bf group and 20.7% in the low_bf group, whereas *Lactobacillus* accounted for 1.9% and 5.5%, respectively (Fig. 1d). At the species level, *Prevotella copri*, *Limosilactobacillus reuteri*, and *Lactobacillus johnsonii* were the three most abundant species across all samples (Fig. 1e).

**Fig. 2:**
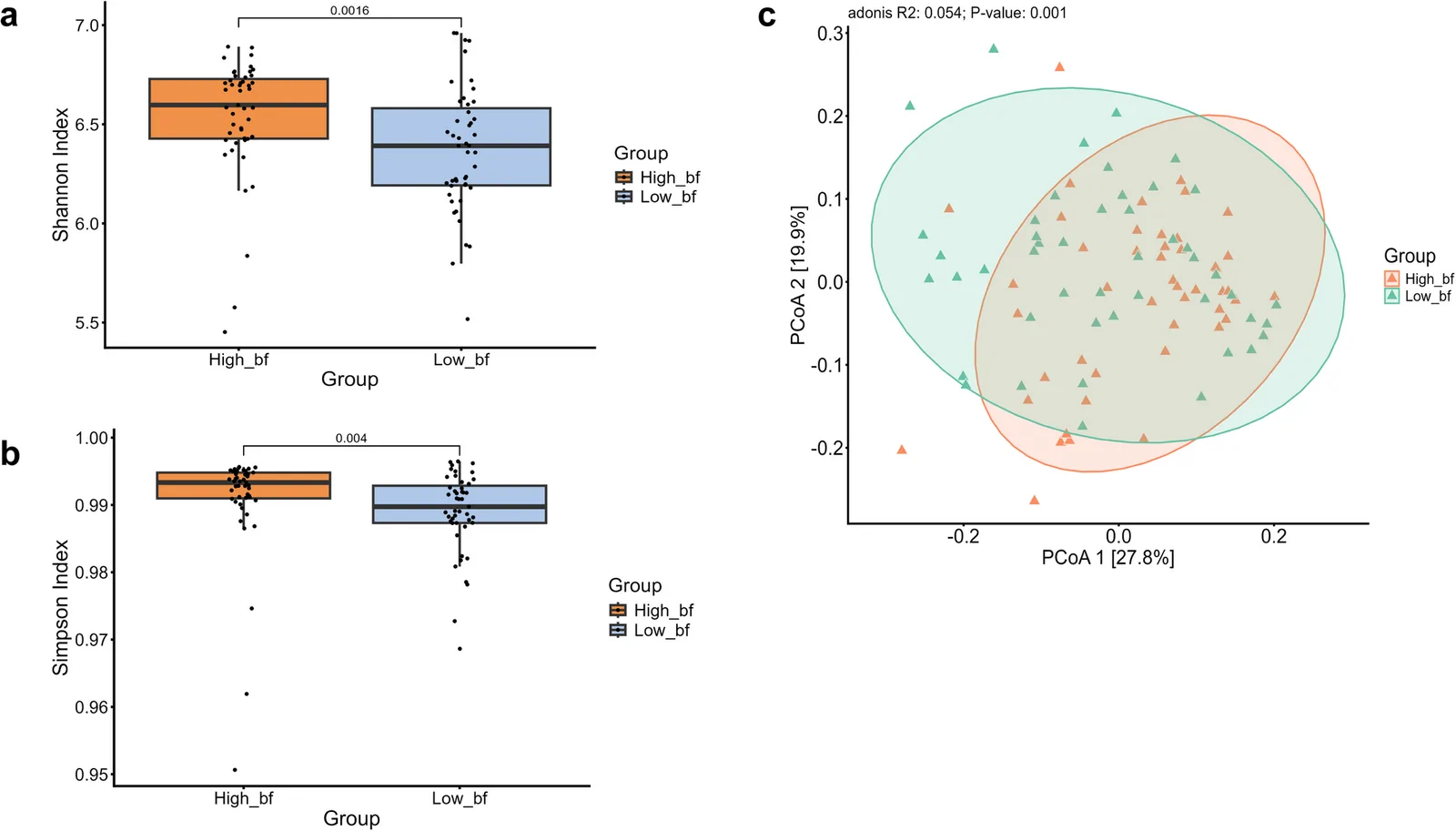
The community diversity between fecal samples from high- and low-fat deposition pigs at the species level. (**a**) The Shannon index in high- and low-bf groups. (**b**) The Simpson index in high- and low-bf groups. (**c**) Principal coordinate analysis (PCoA) of the fecal microbiota. The plot is based on the Bray Curtis distances between pairs of samples. Each point represents one sample; ellipses represent the 95% confidence for all points within each cluster

To assess the alpha-diversity of bacterial communities in pigs with high- and low-fat deposition, we compared the diversity indices (Shannon and Simpson) between the microbiota in high_bf and low_bf groups. We found that high_bf pigs have significantly higher Shannon indices than low_bf pigs (*p*-value < 0.01, Fig. 2a), and the Simpson indices also differed significantly between the two groups (*p*-value < 0.01, Fig. 2b). Beta-diversity was assessed using Bray-Curtis distances and visualized using PCoA (Fig. 2c). Subsequent permutation-based multivariate analysis of variance (PERMANOVA) indicated that species composition differed significantly between the high_bf and low_bf groups, with the group effect explaining 5.44% of the total variation (R² = 0.0544, *p*-value < 0.01).

**Fig. 3:**
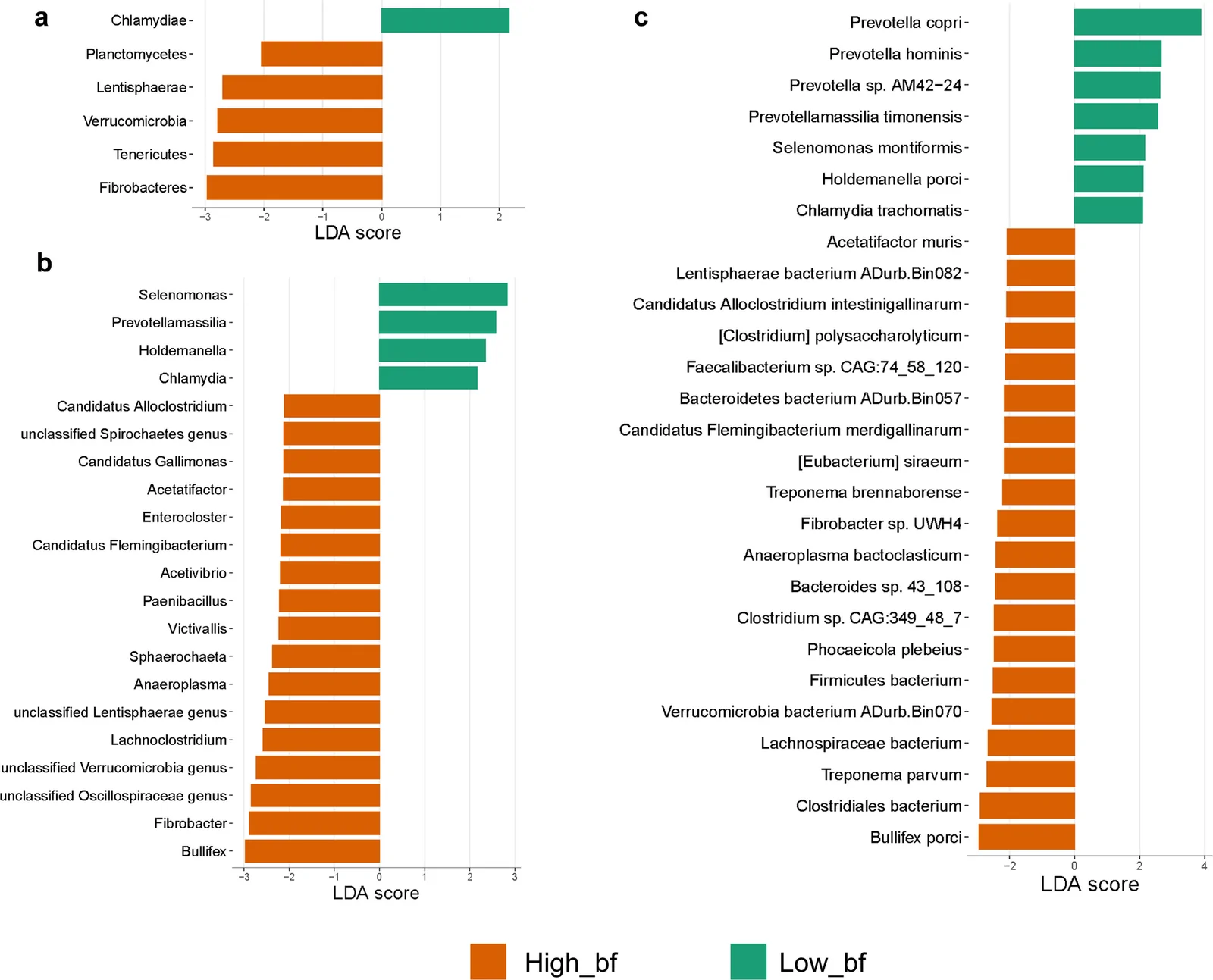
Linear discriminant analysis (LDA) effect size (LEfSe) analysis. The same analysis was performed at the (**a**) phylum, (**b**) genus, and (**c**) species levels to compare the fecal microbiota profiles between the high- and low-fat deposition pigs

To identify specific bacteria with differential abundance between the two groups, we performed LEfSe on the taxa with LDA scores greater than two. At the phylum level, six phyla were characteristic of the respective fat deposition groups, Fibrobacteres, Tenericutes, Verrucomiceobia, Lentisphaerae, and Planctomycetes in the high_bf group, and Chlamydiae in the low_bf group (Fig. 3a). At the genus level, 17 genera in the high_bf group, including *Bullifex*, *Fibrobacter*, *Lachnoclostridium*, and *Anaeroplasma*, and four genera in the low_bf group, including *Selenomonas*, *Prevotellamassilia*, *Holdemanella*, and *Chlamydia*, were identified as potential biomarkers (Fig. 3b). At the species level, seven species in the low_bf group, including three species from the *Prevotella* genus, and 20 species in the high_bf group that included two species from *Treponema* and one species from *Bacteroides* were considered as potential biomarkers for different fat deposition (Fig. 3c; Additional file 2, Table S1). Together, these findings reveal distinct microbial signatures between high- and low-fat deposition pigs, highlighting specific taxa that may influence host energy harvest and fat accumulation.

### Proportion of variation in fat deposition explained by host genetics and gut microbiota

**Fig. 4:**
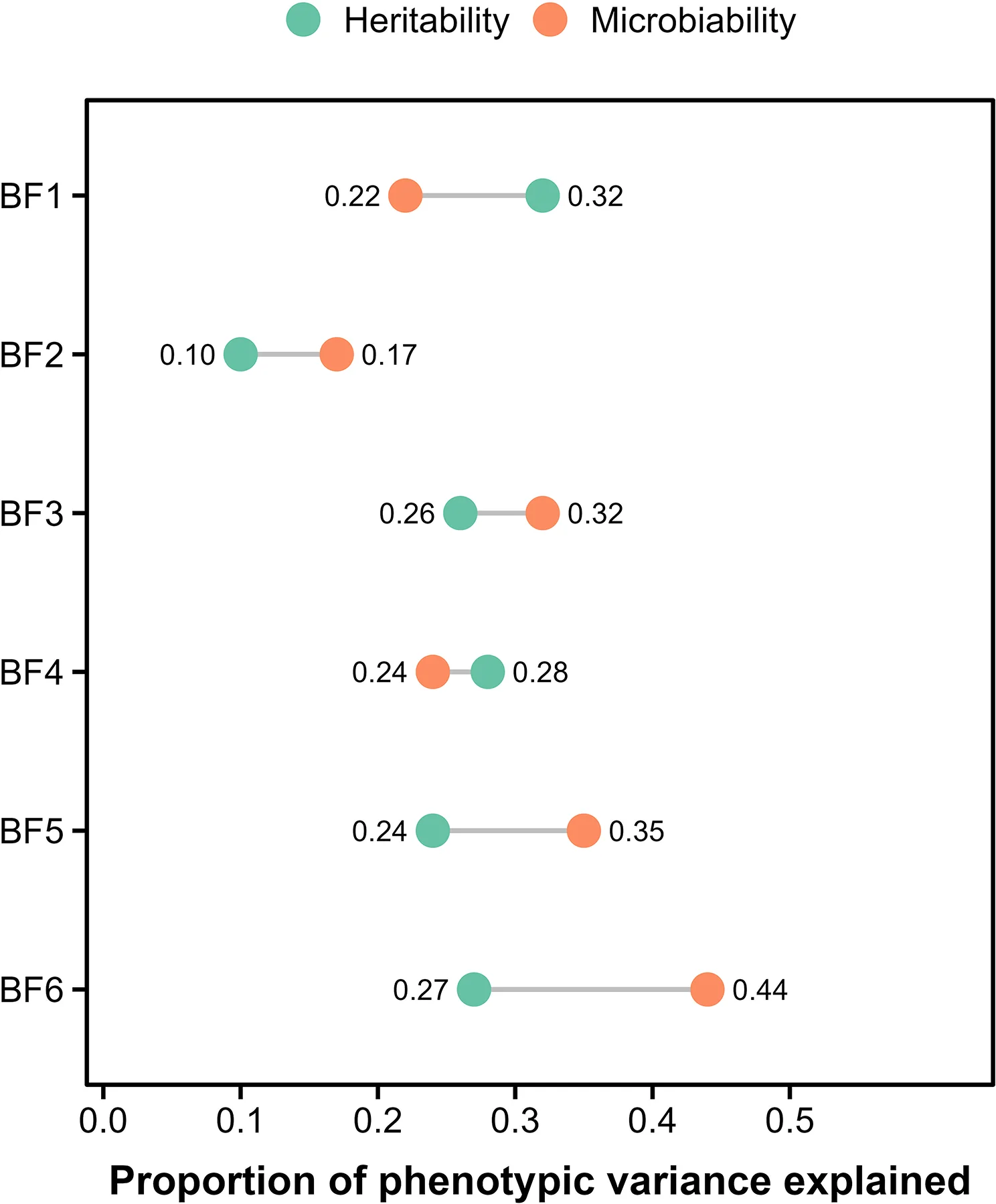
Estimates of heritability and microbiability for fat deposition traits. BF1 represents shoulder backfat thickness; BF2 represents the 6th-7th rib backfat thickness; BF3 represents the last rib backfat thickness; BF4 represents waist backfat thickness; BF5 represents the 3rd-4th last rib backfat thickness; BF6 represents the average backfat thickness

To quantify the contributions of host additive genetics and gut microbiota to fat deposition traits in pigs, we further acquired fecal microbiota profile for all 478 experimental individuals. Host genetics and gut microbiome were as simultaneously modeled as components of the total phenotypic variation, and the heritability and microbiability of six BF traits were estimated. The microbiability estimates for the six BF traits ranged from 0.17 to 0.44, indicating a substantial contribution of the gut microbiota to these traits (Fig. 4).

To investigate whether host genomic similarity was associated with similarity in gut microbiota composition, we performed a Mantel test to examine the associations between the GRM and MRM. A significant correlation was detected between the GRM and MRM (Mantel statistic r: 0.075, *p*-value: 0.001), suggesting that host genetic relatedness was associated with overall microbiome similarity. However, this matrix-level correspondence does not directly indicate that host genetic effects interact with microbiome effects to influence phenotypic variation. To further evaluate whether this correspondence contributed to phenotypic variation through interaction effects, we included an interaction term in an expanded model (Model C). The results showed that the host genomic and microbiome effects for the six traits remained largely consistent with those from Model B (Table 3). The variance explained by the interaction effect accounted for only a minor fraction (0.1 ~ 0.6%) of the total phenotypic variance, suggesting that we did not detect a significant interaction effect between the host genome and gut microbiome contributing to fat deposition traits in the present population.

**Table 3:** Comparison of variance component estimates for fat deposition traits across different models

Traits	Model	$\:{\boldsymbol{\sigma\:}}_{\boldsymbol{g}}^{2}$(SE)	$\:{\boldsymbol{\sigma\:}}_{\boldsymbol{g}}^{2}/{\boldsymbol{\sigma\:}}_{\boldsymbol{p}}^{2}$(SE)	$\:{\boldsymbol{\sigma\:}}_{\boldsymbol{m}}^{2}$(SE)	$\:{\boldsymbol{\sigma\:}}_{\boldsymbol{m}}^{2}/{\boldsymbol{\sigma\:}}_{\boldsymbol{p}}^{2}$(SE)	$\:{\boldsymbol{\sigma\:}}_{\boldsymbol{i}}^{2}$(SE)	$\:{\boldsymbol{\sigma\:}}_{\boldsymbol{i}}^{2}/{\boldsymbol{\sigma\:}}_{\boldsymbol{p}}^{2}$(SE)
BF1	Model A	0.16(0.06)	0.38(0.14)				
	Model B	0.14(0.06)	0.32(0.13)	0.09(0.05)	0.22(0.12)		
	Model C	0.14(0.06)	0.32(0.12)	0.10(0.05)	0.22(0.12)	0.002(0.099)	0.004(0.225)
BF2	Model A	0.05(0.04)	0.13(0.12)				
	Model B	0.04(0.05)	0.10(0.11)	0.07(0.05)	0.17(0.11)		
	Model C	0.04(0.05)	0.10(0.11)	0.07(0.05)	0.17(0.12)	0.001(0.086)	0.003(0.218)
BF3	Model A	0.07(0.03)	0.36(0.13)				
	Model B	0.06(0.03)	0.26(0.12)	0.07(0.03)	0.32(0.14)		
	Model C	0.06(0.03)	0.26(0.12)	0.07(0.03)	0.32(0.14)	0.001(0.047)	0.004(0.219)
BF4	Model A	0.09(0.03)	0.33(0.13)				
	Model B	0.08(0.04)	0.28(0.12)	0.07(0.04)	0.24(0.14)		
	Model C	0.08(0.04)	0.28(0.12)	0.07(0.05)	0.24(0.15)	0.002(0.064)	0.006(0.223)
BF5	Model A	0.07(0.03)	0.29(0.13)				
	Model B	0.06(0.03)	0.24(0.12)	0.09(0.04)	0.35(0.14)		
	Model C	0.06(0.03)	0.24(0.12)	0.09(0.05)	0.35(0.15)	0.000(0.061)	0.001(0.230)
BF6	Model A	0.08(0.03)	0.37(0.13)				
	Model B	0.06(0.03)	0.27(0.11)	0.10(0.04)	0.44(0.14)		
	Model C	0.06(0.03)	0.27(0.11)	0.10(0.04)	0.44(0.16)	0.001(0.053)	0.003(0.225)

BF1 represents shoulder backfat thickness; BF2 represents the 6th-7th rib backfat thickness; BF3 represents the last rib backfat thickness; BF4 represents waist backfat thickness; BF5 represents the 3rd-4th last rib backfat thickness; BF6 represents the average backfat thickness. Model A included the host genomic effect; Model B included both host genomic and microbiome effects; Model C further included the interaction effect between the host genome and the gut microbiome. $\:{\sigma\:}_{g}^{2}$ represents the genetic variance, $\:{\sigma\:}_{g}^{2}/{\sigma\:}_{p}^{2}$ represents the proportion of genetic variance to the phenotypic variance, which is the heritability, and SE is the standard error; $\:{\sigma\:}_{m}^{2}$represents the variance of the gut microbiome, $\:{\sigma\:}_{m}^{2}/{\sigma\:}_{p}^{2}$represents the proportion of the gut microbiome variance to the phenotypic variance, which is the microbiability; $\:{\sigma\:}_{i}^{2}$ represents the variance of the interaction effect between host genome and the gut microbiome, $\:{\sigma\:}_{i}^{2}/{\sigma\:}_{p}^{2}$ represents the proportion of the interaction effect variance to the phenotypic variance

### Phenotypic prediction of fat deposition traits using genomic and microbiome information

**Fig. 5:**
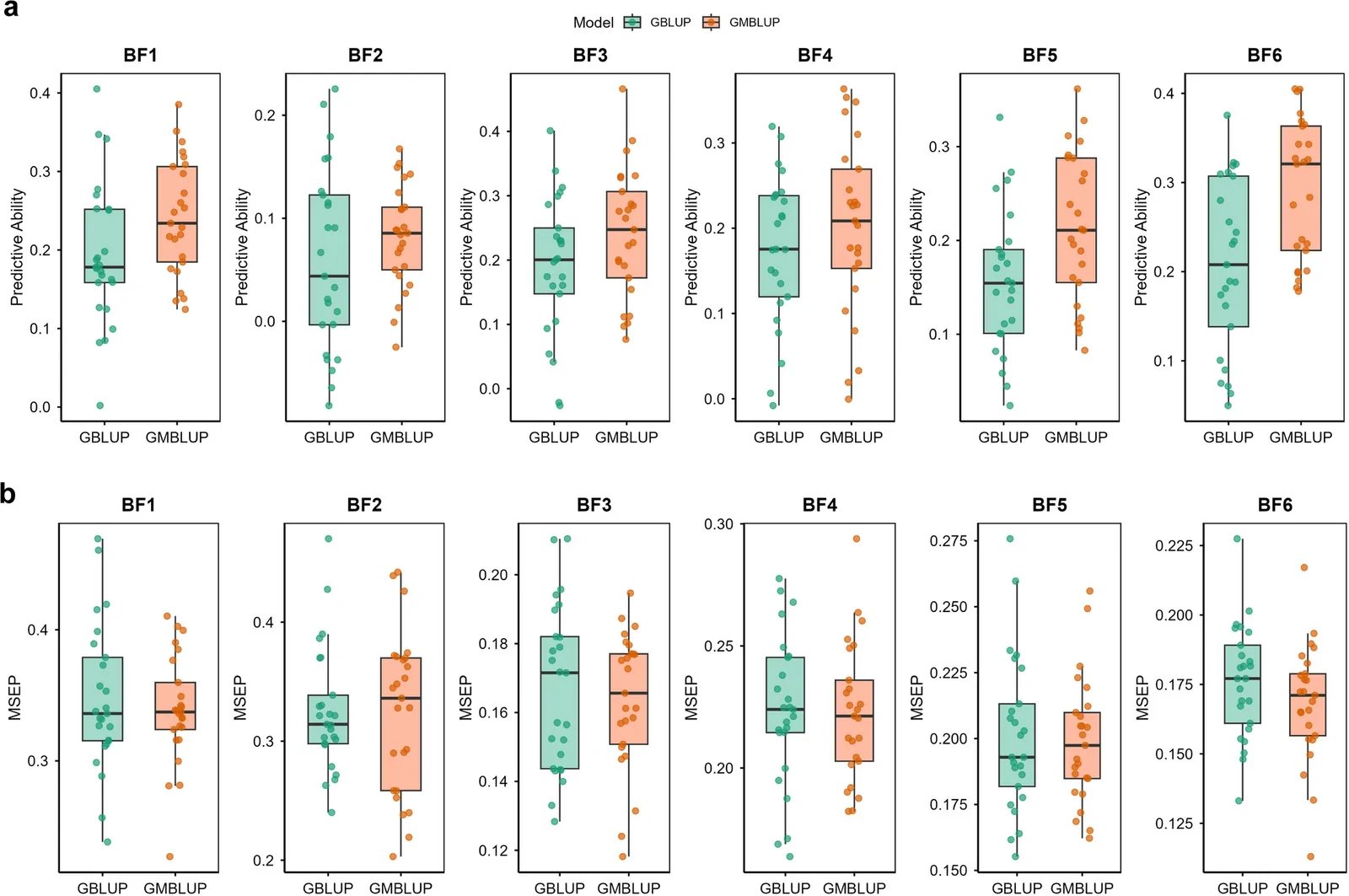
Prediction performance of GBLUP and GMBLUP for six fat deposition traits. (**a**) Distribution of predictive ability across 5 × 5 validation folds. (**b**) Distribution of mean-squared error of prediction (MSEP) across 5 × 5 validation folds. Each point represents one validation fold from five replicates of five-fold cross-validation and horizontal lines indicate the median. GBLUP represents the model using host genomic information, whereas GMBLUP represents the model jointly incorporating host genomic and gut microbial information. BF1 represents shoulder backfat thickness; BF2 represents the 6th -7th rib backfat thickness; BF3 represents the last rib backfat thickness; BF4 represents waist backfat thickness; BF5 represents the 3rd -4th last rib backfat thickness; BF6 represents the average backfat thickness

Based on whole-genome sequencing and metagenomic data, we evaluated whether integrating host genomic and microbiome information could improve the phenotypic prediction of BF traits. Under the standard GBLUP model, the mean predictive abilities for six traits ranged from 0.059 to 0.208, with median values ranging from 0.044 to 0.208 (Additional file 2, Table S2). When both genomic and microbiome information were incorporated into the model, GMBLUP achieved mean predictive abilities of 0.242, 0.082, 0.241, 0.201, 0.213, and 0.292 for BF1 to BF6, respectively, with corresponding median values of 0.234, 0.086, 0.247, 0.209, 0.211, and 0.321 (Additional file 2, Table S2). Compared with GBLUP, GMBLUP increased the mean predictive ability by 0.023 to 0.084 across traits, corresponding to relative increases of 24.7%, 38.9%, 26.2%, 14.2%, 36.5%, and 40.3% for BF1 to BF6, respectively (Fig. 5a; Additional file 2, Table S2). The magnitude of improvement varied among traits, with the largest increase observed for BF6. To further evaluate prediction error, we calculated the mean squared error of prediction for each validation fold. The mean MSEP ranged from 0.167 to 0.347 under GBLUP and from 0.164 to 0.339 under GMBLUP. The corresponding median MSEP values ranged from 0.172 to 0.336 for GBLUP and from 0.166 to 0.337 for GMBLUP. Compared with GBLUP, GMBLUP slightly reduced the mean MSEP by 0.001 to 0.009 across the six traits (Fig. 5b; Additional file 2, Table S2). These results suggest that incorporating microbiome information modestly improved phenotypic predictive ability and slightly reduced prediction error for BF traits.

## Discussion

Fat deposition is a complex quantitative trait that is influenced by both genetics and the environment. With the advancement of microbiome research, the gut microbiome has been shown to explain part of the phenotypic variation of complex traits in many different livestock species [42–44]. Based on metagenomic sequencing, we revealed compositional differences in the gut microbiota between pigs with high- and low-fat deposition. Firmicutes and Bacteroidetes were the dominant phyla in the fecal microbiota, together accounting for an average of 83.9% of the total abundance, which is consistent with previous studies [45, 46]. Additionally, Spirochaetes and Proteobacteria were also major components of the fecal microbiota in DLY pigs. At the genus level, the ten most abundant taxa were largely similar between the two groups, with *Prevotella*, *Treponema*, *Bacteroides*, and *Lactobacillus* ranking among the most prevalent. It should be noted, however, that the predominant genera can vary across pig breeds, as reported [47], likely due to genetic and environmental differences. Previous research on the porcine gut microbiota has largely utilized 16S rRNA gene sequencing due to its lower cost, however, this approach typically provides taxonomic resolution only to the genus level. In contrast, metagenomic sequencing captures full genomic content, allowing for more precise identification of microbial taxa at the species or even strain level. Leveraging this method, we identified several bacterial species of interest in DLY pigs, such as *Prevotella copri*, *Limosilactobacillus reuteri*, and *Lactobacillus johnsonii*. Notably, *Lactobacillus johnsonii* has been implicated in sugar transport and metabolism and contributes to the maintenance of intestinal homeostasis [48]. To further identify potential biomarkers associated with fat deposition in DLY pigs, LEfSe identified 27 bacterial species with differential abundance between the high_bf and low_bf groups. Several *Prevotella*-related species, including *Prevotella copri*, *Prevotella hominis*, and *Prevotella sp. AM42-24*, were enriched in the low_bf group. *Prevotella* species are common members of the pig gut microbiota and are involved in carbohydrate fermentation and fermentation-derived metabolite production [49]. *P. copri* has been reported to be negatively associated with adiposity in human and mouse studies [50], whereas a study in Duroc pigs reported that *P. copri* was associated with increased fat accumulation under formula diets [51]. These inconsistent findings indicate that the relationship between *P. copri* and fat deposition may be context-dependent. In the high_bf group, several enriched species belonged to taxa related to complex carbohydrate degradation or fiber utilization, including Treponema and Bacteroides-related species. Treponema has been associated with fiber digestibility in pigs, and Bacteroides-related taxa have broad capacities for dietary polysaccharide utilization [52, 53]. These taxa may reflect differences in microbial substrate utilization between pigs with divergent fat deposition levels. Nevertheless, the identified species should be regarded as fat-deposition-associated microbial features rather than causal biomarkers. Further microbial functional profiling and experimental validation are needed to determine whether these species directly contribute to fat accumulation in pigs.

Microbiability provides a quantitative measure of the contribution of the gut microbiome to phenotypic variation in fat deposition traits. A related human study later referred to this measure as the microbiome association index and demonstrated that it can be reliably estimated even with sample sizes of only several hundred individuals [54]. In the present study, metagenomic sequencing data from fecal samples of DLY pigs were used to construct the MRM among individuals based on species level relative abundances. A linear mixed model accounting for both host genetic effects and microbiome effects was applied to estimate microbiability for six fat deposition traits, yielding values of 0.17 to 0.44. These results indicate that the gut microbiome accounts for a certain proportion of phenotypic variation. Higher microbiability estimates suggest a greater microbiome contribution to trait variation, suggesting that gut microbiome information contributes to explaining variation in fat deposition traits. Estimates from previous studies based on microbial communities from different intestinal segments have ranged from 0.11 to 0.31 [55]. While studies in Tongcheng × Large White pigs reported microbiability of 0.11 ~ 0.20 for backfat thickness, slightly below the heritability of the trait [56]. In contrast, He et al. [57] observed a microbiability estimate of 0.42. Differences among studies may reflect variation in breed, management conditions, sampling location within the gut, and sequencing methodology. Additionally, the construction of the MRM requires careful consideration because microbiome relative abundance data are compositional. Different transformation and scaling strategies for microbiome abundance data may emphasize different aspects of microbial community variation and, consequently, may affect the estimated microbiome-based relationships among individuals. Therefore, comparisons of microbiability across studies should be interpreted with caution. Most previous studies have relied on OTU-based microbiome profiles, which offer lower taxonomic resolution than metagenomic data. By leveraging species level information, our study provides empirical estimates of microbiability for fat deposition traits in pigs based on the metagenomic data.

The above were only focused on single effects, that is to say only the independent influence of host genome or the microbiota. However, whether the host genetics may interact with the gut microbiota to influence fat deposition in pigs has remained largely unknown. Considering the potential influence of host genetics on the gut microbiome, we incorporated interaction effects between the host genome and the microbiome into the mixed model to evaluate their contribution to phenotypic variation. The results indicate that the variance explained by these interaction effects was close to zero, which is consistent with previous reports [56]. The relatively limited sample size of the present study may have reduced the ability to detect small interaction effects. Consequently, the absence of a statistically supported interaction effect in this dataset does not demonstrate that such interactions are biologically absent. Larger populations will be required to evaluate interactions more reliably. The Mantel test is commonly employed in microbiome research to assess correlations between host genetics and gut microbiome composition. In this study, a significant association was detected between the host genomic kinship matrix and the gut microbiota relationship matrix (*r* = 0.075), consistent with observations in human [58] and mice [59]. Nevertheless, the Mantel test has inherent limitations when used to evaluate the influence of host genetics on the overall microbiome structure. As a matrix-based approach, it primarily captures linear relationships and may overlook nonlinear interactions. Additionally, the gut microbiota exhibits high complexity and interactions among them, which may affect the observed correlation strength. Therefore, although the correlation detected was weak, it suggests a limited association between host genetic background and gut microbiome composition.

Advances in animal breeding rely partly on the integration of new predictive indicators in genomic selection to improve accuracy for complex traits [60]. Beyond genomic data, microbiome abundance profiles also serve as an informative source for predicting complex traits in pigs. Previous studies have indicated that prediction models based solely on microbiome data can outperform traditional genomic prediction models [18, 61]. However, most existing research has been limited to either host genomic or microbiome data alone, lacking systematic integration of both omics layers. To address this, the present study integrated host genomic and gut metagenomic information within a prediction model and evaluated predictive ability and MSEP for fat deposition traits in pigs. Across the six BF traits, GMBLUP showed numerically higher mean predictive abilities than GBLUP, with absolute increases ranging from 0.023 to 0.084. The MSEP was also numerically lower under GMBLUP, with reductions ranging from 0.001 to 0.009. Although the absolute increases in predictive ability were modest, the corresponding relative increases were comparatively substantial for some traits. However, these differences were not statistically significant. Thus, GMBLUP showed modest numerical gains in predictive ability but did not provide a statistically significant improvement over GBLUP in the present population. These results suggest that microbiome information may provide complementary predictive information beyond host genomic data, although its contribution to prediction accuracy was limited under the current population size and prediction framework. The GO-BLUP model proposed by Christensen [23] was developed for genetic evaluation with intermediate omics features, in which the breeding value is decomposed into an omics-mediated breeding value and a residual breeding value. This framework provides a more formal approach for incorporating omics information into genetic evaluation. In contrast, the GMBLUP model used in the present study fitted the microbial relationship matrix as an additional random effect, and the combined genomic and microbiome effects were used to evaluate phenotypic predictive ability. Therefore, the microbiome effect estimated by GMBLUP should not be interpreted directly as a breeding value because it may include both host-genetically controlled and non-genetic microbiome variation. The contribution of microbiome information to genetic evaluation depends not only on its association with the phenotype but also on the extent to which the microbiome effect is controlled by host genetics. Although the animals used in this study were commercial crossbred pigs rather than individuals from a nucleus breeding population, the primary objective of this study was to integrate host whole-genome resequencing data with gut metagenomic sequencing data to characterize host-microbiome contributions to fat deposition traits and to evaluate the feasibility of microbiome-assisted phenotypic prediction.

The composition of the gut microbiota in pigs is not static but undergoes dynamic changes from birth to adulthood, reaching a relatively stable state during the nursery period [62]. In this study, to minimize heterogeneity in gut microbiome composition, we collected fecal samples for microbiome profiling from pigs reared under standardized conditions at a market weight of approximately 110 kg. Whether the early-life gut microbiota of piglets can serve as a reliable predictor for complex host traits remains an open question for future investigation. In addition, although metagenomic sequencing provides information on both microbiome taxonomic composition and functional potential, the present study focused primarily on species-level microbiome composition. Future studies that integrate longitudinal microbiome sampling with functional genes and taxonomic profiles may provide a more comprehensive understanding of the biological mechanisms linking gut microbiota to complex economic traits.

## Conclusion

In conclusion, fecal microbial community structure differed among DLY pigs with contrasting fat deposition phenotypes, and 27 species were identified as potential biomarkers associated with fat deposition. Estimates of heritability and microbiability indicated that variation in fat deposition traits was associated with both host genomic and gut microbiome effects, whereas the genomic-by-microbiome interaction explained only a negligible proportion of phenotypic variation. The absolute improvement of GMBLUP over GBLUP was modest, with small increases in predictive ability and slight reductions in prediction error across the six fat deposition traits. Although GMBLUP consistently showed numerically higher predictive ability, these differences were not statistically significant, indicating no statistically significant improvement over GBLUP in the present population. Nevertheless, the numerical gains suggest that gut microbiome information may have complementary predictive value beyond host genomic data, although its contribution to prediction was limited under the current population size and prediction framework.

## Supplementary Information

Below is the link to the electronic supplementary material.

Supplementary Material 1

Supplementary Material 2

## Data Availability

The data that support the findings of this study are included in the main paper and supplementary materials.
