# Supplementary material for: Integrating whole-genome sequencing and metagenomics improves phenotype prediction of fat deposition traits in pigs
Source: Genet Sel Evol. 2026 Nov 13;58:90. doi: 10.1186/s12711-026-01116-y (PMC13760891; doi:10.1186/s12711-026-01116-y)
Supplement: Supplementary file 1 [file 12711_2026_1116_MOESM1_ESM.docx]

**Additional file 1**

**Figure S1** **The distribution of six fat deposition phenotypes.**

BF1 represents shoulder backfat thickness; BF2 represents the 6th-7th rib backfat thickness; BF3 represents the last rib backfat thickness; BF4 represents waist backfat thickness; BF5 represents the 3rd-4th last rib backfat thickness; BF6 represents the average backfat thickness.
