# Supplementary material for: Integrating whole-genome sequencing and metagenomics improves phenotype prediction of fat deposition traits in pigs
Source: Genet Sel Evol. 2026 Nov 13;58:90. doi: 10.1186/s12711-026-01116-y (PMC13760891; doi:10.1186/s12711-026-01116-y)
Supplement: Supplementary file 2 [file 12711_2026_1116_MOESM2_ESM.docx]

**Additional file 2**

**Table S1 The biomarker species between the high-fat and low-fat deposition groups.**

| **Species** | **Group** | **LDA（log10）** | ***P*.adjust** |
| --- | --- | --- | --- |
| *Prevotella copri* | Low_bf | 3.919 | 2.19E-02 |
| *Bullifex porci* | High_bf | 2.938 | 3.01E-02 |
| *Clostridiales bacterium* | High_bf | 2.899 | 4.03E-02 |
| *Lachnospiraceae bacterium* | High_bf | 2.693 | 2.03E-02 |
| *Prevotella hominis* | Low_bf | 2.691 | 3.29E-02 |
| *Treponema parvum* | High_bf | 2.673 | 4.80E-02 |
| *Prevotella sp. AM42-24* | Low_bf | 2.663 | 4.14E-02 |
| *Verrucomicrobia bacterium ADurb.Bin070* | High_bf | 2.579 | 1.15E-02 |
| *Prevotellamassilia timonensis* | Low_bf | 2.554 | 2.40E-02 |
| *Firmicutes bacterium* | High_bf | 2.496 | 3.71E-02 |
| *Phocaeicola plebeius* | High_bf | 2.488 | 1.32E-02 |
| *Anaeroplasma bactoclasticum* | High_bf | 2.442 | 1.14E-02 |
| *Clostridium sp. CAG:349_48_7* | High_bf | 2.438 | 3.48E-02 |
| *Bacteroides sp. 43_108* | High_bf | 2.412 | 1.64E-02 |
| *Fibrobacter sp. UWH4* | High_bf | 2.347 | 2.83E-02 |
| *Selenomonas montiformis* | Low_bf | 2.235 | 5.05E-03 |
| *Treponema brennaborense* | High_bf | 2.185 | 3.80E-03 |
| *[Eubacterium] siraeum* | High_bf | 2.180 | 1.14E-02 |
| *Bacteroidetes bacterium ADurb.Bin057* | High_bf | 2.167 | 2.54E-02 |
| *Candidatus Flemingibacterium merdigallinarum* | High_bf | 2.156 | 8.33E-03 |
| *Acetatifactor muris* | High_bf | 2.117 | 6.64E-03 |
| *Faecalibacterium sp. CAG:74_58_120* | High_bf | 2.112 | 4.47E-03 |
| *[Clostridium] polysaccharolyticum* | High_bf | 2.100 | 6.71E-03 |
| *Chlamydia trachomatis* | Low_bf | 2.098 | 1.18E-02 |
| **Species** | **Group** | **LDA（log10）** | ***P*.adjust** |
| *Holdemanella porci* | Low_bf | 2.090 | 3.67E-02 |
| *Candidatus Alloclostridium intestinigallinarum* | High_bf | 2.068 | 1.89E-02 |
| *Lentisphaerae bacterium ADurb.Bin082* | High_bf | 2.058 | 1.42E-02 |

**Table S2 The phenotypic prediction of fat deposition traits using GBLUP and GMBLUP models.**

| **Trait** | **Model** | **Predictive ability** | | **MSEP** | |
| --- | --- | --- | --- | --- | --- |
|  |  | **Mean ± SE** | **median** | **Mean ± SE** | **median** |
| BF1 | GBLUP | 0.194 ± 0.018 | 0.178 | 0.347 ± 0.011 | 0.336 |
|  | GMBLUP | 0.242 ± 0.015 | 0.234 | 0.339 ± 0.008 | 0.337 |
| BF2 | GBLUP | 0.059 ± 0.018 | 0.044 | 0.326 ± 0.011 | 0.314 |
|  | GMBLUP | 0.082 ± 0.014 | 0.086 | 0.324 ± 0.013 | 0.336 |
| BF3 | GBLUP | 0.191 ± 0.021 | 0.200 | 0.167 ± 0.005 | 0.172 |
|  | GMBLUP | 0.241 ± 0.020 | 0.247 | 0.164 ± 0.004 | 0.166 |
| BF4 | GBLUP | 0.176 ± 0.018 | 0.175 | 0.223 ± 0.006 | 0.224 |
|  | GMBLUP | 0.201 ± 0.021 | 0.209 | 0.223 ± 0.006 | 0.221 |
| BF5 | GBLUP | 0.156 ± 0.015 | 0.154 | 0.201 ± 0.006 | 0.193 |
|  | GMBLUP | 0.213 ± 0.016 | 0.211 | 0.199 ± 0.005 | 0.197 |
| BF6 | GBLUP | 0.208 ± 0.019 | 0.208 | 0.176 ± 0.004 | 0.177 |
|  | GMBLUP | 0.292 ± 0.016 | 0.321 | 0.169 ± 0.004 | 0.171 |

BF1 represents shoulder backfat thickness; BF2 represents the 6th–7th rib backfat thickness; BF3 represents the last rib backfat thickness; BF4 represents waist backfat thickness; BF5 represents the 3rd–4th last rib backfat thickness; BF6 represents the average backfat thickness.
